# The Effect of TGFβ1 in Adipocyte on Inflammatory and Fibrotic Markers at Different Stages of Adipocyte Differentiation

**DOI:** 10.3390/pathophysiology29040050

**Published:** 2022-11-23

**Authors:** Babu Raja Maharjan, Susan V. McLennan, Stephen M. Twigg, Paul F. Williams

**Affiliations:** 1 Greg Brown Diabetes & Endocrinology Laboratory, Sydney Medical School, University of Sydney, Sydney, NSW 2006, Australia; 2 School of Medicine, Department of Biochemistry, Patan Academy of Health Sciences, Lalitpur 44700, Nepal; 3 New South Wales Health Pathology, Sydney, NSW 2050, Australia; 4 Department of Endocrinology, Royal Prince Alfred Hospital, Sydney, NSW 2006, Australia

**Keywords:** TGFβ, adipocyte, stages of differentiation, inflammation, fibrosis

## Abstract

Transforming growth factor beta (TGFβ) is a versatile cytokine. Although a profibrotic role of TGFβ is well established, its effect on tissue inhibitor of metalloproteinase (TIMPs) and inflammatory mediators are incompletely described. This study investigates the profibrotic and pro-inflammatory role of TGFβ1 during adipocyte differentiation. NIH3T3L1 cells were used for the in vitro study and were differentiated by adding a standard differentiation mix either with rosiglitazone (R-Diff) or without (S-Diff). Recombinant TGFβ1 (2 ng/mL) was added to the undifferentiated preadipocyte during the commitment stage and at the terminal differentiation stage. TGFβ1 treatment significantly decreased adiponectin mRNA at both early commitment (>300 fold) and terminal differentiated cells [S-Diff (~33%) or R-Diff (~20%)]. TGFβ1 upregulated collagen VI mRNA and its regulators connective tissue growth factor (CCN2/CTGF), TIMP1 and TIMP3 mRNA levels in undifferentiated preadipocytes and adipocytes at commitment stage. But in the terminal differentiated adipocytes, changes in mRNA and protein of collagen VI and TIMP3 mRNA were not observed despite an increase in CCN2/CTGF, TIMP1 mRNA. Although TGFβ1 upregulated interleukin-6 (IL6) and monocyte chemoattractant protein-1 (MCP1) mRNA at all stages of differentiation, decreased tumor necrosis factor-α (TNFα) mRNA was observed early in adipocyte differentiation. This study highlights the complex role of TGFβ1 on extracellular matrix (ECM) remodeling and inflammatory markers in stimulating both synthetic and inhibitory markers of fibrosis at different stages of adipocyte differentiation.

## 1. Introduction

Transforming growth factor beta 1 (TGFβ1) is associated with obesity and insulin resistance in both animal and human models [1,2]. Insulin and high glucose are known to stimulate the recruitment of TGFβ1 receptors to the cell surface [3]. In addition, the circulating TGFβ levels are significantly elevated in obese humans, ob/ob mice, and obese mice fed a High Fat Diet (HFD) [2,4]. TGFβ1 is constitutively expressed in adipose tissue in vivo, in cultured preadipocytes and in differentiated adipocytes [5,6]. TGFβ signaling regulates many cellular processes such as autophagy, apoptosis, inflammation, fibrosis, and adipocyte differentiation [7].

TGFβ has the ability to promote or suppress cellular proliferation depending on the cell types and state of differentiation to which TGFβ was added [8,9,10]. Since adipose tissue contains adipocytes at different stages of maturation [5,6], the effect of TGFβ may be difficult to determine in a mixed population. Our earlier study confirmed that during adipocyte differentiation, there were differential effects of TGFβ1 on thermogenic makers depending upon the adipocyte maturation [11]. Although the effect of TGFβ1 on collagen VI, connective tissue growth factor (CTGF), tissue inhibitor of metalloproteinase-1 (TIMP1), and inflammatory markers in adipocytes and other tissues have been described [12,13,14,15,16,17], studies defining the effect of TGFβ on these markers during adipocyte differentiation are rare. The unique part of this study describes an as-yet unreported effect of TGFβ1 on the tissue inhibitor of metalloproteinase-3 (TIMP3), an important anti-fibrotic regulator of extracellular matrix (ECM) remodeling in adipocytes. Therefore, we have explored the effect of TGFβ1 on ECM remodeling, inflammatory markers, and TIMP3 during adipocyte differentiation in 3T3L1 cells.

## 2. Materials and Methods

### 2.1. Culture and Differentiation of NIH3T3L1 Cells

NIH 3T3L1 cells (obtained from American Type Culture Collection) were cultured in Dulbecco’s Modified Eagles Medium containing 25 mM glucose (DMEM/high glucose), Fetal Calf Serum (FCS, 10%), and Penicillin/Streptomycin (P/S, 1%) in an incubator at 37 °C with 5% CO_2_. A differentiation mix was added to the fibroblast after obtaining a confluence of 95–100%. Standard differentiation mix (S-Diff) contains 1 μg/mL insulin, 1 μmol/L dexamethasone, and 115 μg/mL 3-isobutyl-1-methylxanthine (IBMX). Since rosiglitazone has been shown to stimulate adipocyte differentiation, S-Diff supplemented with rosiglitazone (1 μg/mL) was also used as a rosiglitazone-supplemented differentiation mix (R-Diff) [18]. The changes in media were done on day 3, day 6, and day 8 with DMEM/high glucose containing FCS, Penicillin/Streptomycin that had been supplemented with insulin (1 μg/mL).

### 2.2. TGFβ1 Treatment

In order to study the effect of the TGFβ1 on different phases of adipocyte differentiation (preadipocyte, commitment, and terminal differentiation), recombinant active human (rh) TGFβ1 protein (2 ng/mL: Catalogue Number 240-B-010, R & D Systems, Minneapolis, MN, USA) was used. This dose has been shown previously and in the present study (data not shown) to upregulate fibronectin gene expression by 6-fold compared with untreated cells [19].

As described previously [11], to investigate the effect of TGFβ1 on preadipocyte, rhTGFβ1 (2 ng/mL) with 10% FCS in DMEM was added at day 0 to confluent NIH 3T3L1 fibroblasts without differentiation mix. In order to observe the effect of TGFβ1 on the commitment stage of adipocyte differentiation, rhTGFβ1 (2 ng/mL) was added at day 0 to confluent NIH 3T3L1 fibroblasts in 10% FCS in DMEM with either a standard differentiation mix or one with added Rosiglitazone. Both preadipocytes and adipocytes at the commitment stage were harvested after 3 days.

In order to investigate the effect of TGFβ1 on mature adipocytes, the media were changed at day 10 to DMEM containing 0.1% BSA, and 12 h later, the mature adipocytes were treated with rhTGFβ1 (2 ng/mL) in DMEM and 0.1% BSA. Mature adipocytes were harvested after 2 days. Confluent un-differentiated NIH 3T3L1 cells (Un-Diff) acted as a control. For all studies, during the cell harvest, the cells were washed twice with PBS (2 mL) in a culture plate and then collected for analysis of gene and protein markers for adipogenesis.

### 2.3. RNA Extraction

The mRNA levels of various markers for adipocyte differentiation, ECM remodeling, and inflammation (Table 1) were measured by qRT-PCR in the NIH3T3L1 cells as described previously [11]. Briefly, the RNA was extracted using TRI reagent (Sigma, Darmstadt, Germany), washed in 70% ethanol dried, resuspended in 20 μL of RNase-free water, and quantitated using the Nanodrop and stored at −80 °C for future use. RNA (1 μg) was reverse transcribed using 50 pmol of oligo(dT)12–18 (Life Technologies, Carlsbad, CA, USA) and 0.4 pmol of random hexamers (Life Technologies) at 70 °C for 10 min then added 10 mM of DTT (Life Technologies), 0.05 mM of dNTPs (Bioline, London, UK) and l00 U of superscript (Life Technologies, CA, USA) in PCR machine (BioRad, Hercules, CA, USA) that was programmed as described previously [11]. The mRNA levels were calculated using Delta/Delta method with NoNo used as the reference gene and results were expressed as fold change relative to control reference gene levels.

### 2.4. Protein Extraction and Quantification

The protein levels were measured by Western immunoblot in cells solubilized in RIPA buffer (100 μL) containing a cOmplete™ protease inhibitor cocktail (Roche, Rotkreuz, Switzerland), as explained earlier [11]. Anti-collagen VI antibody (catalog number ab6588, Abcam, Cambridge, UK) and anti-rabbit IgG for the secondary antibody (catalog number S9169, Sigma^®^) were diluted at 1:500 and 1:10,000, respectively. Quantification of protein was undertaken by normalizing the size of the band of protein of interest against the total protein load quantified using the stain-free technique and ImageLabSoftware V4.1 (Bio-Rad, Hercules, CA, USA) [20,21].

### 2.5. Oil Red O Staining

As described previously [11], the cells were stained with Oil Red O (ORO) on day 10 to track the differentiation of the 3T3L1 fibroblast cells into fat cells. Images were obtained using an Olympus microscope at 10× objective.

### 2.6. Statistical Analysis

The data collected from the various experiments were entered into the Prism Graph pad 7. We used one-way ANOVA with Tukey’s multiple comparison test and two-way ANOVA with Sidak’s multiple comparison test as appropriate. Data were mainly expressed as Mean ± SD with the symbol indicating statistical significance at *p* < 0.05.

## 3. Results

### 3.1. Changes in Markers at Different Stages of Adipocyte Differentiation

**Figure 1:**
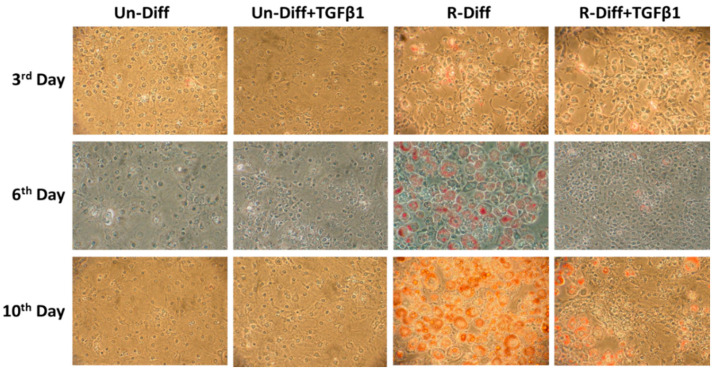
Effect of TGFβ1 on adipocyte differentiation on undifferentiated preadipocytes and early commitment stage of adipocyte differentiation. TGFβ1 was added to cells at day 0. A representative picture at 100× magnification was taken on days 3, 6, and 10 after Oil Red O staining for TGFβ1 treatment on undifferentiated preadipocytes (Un-Diff) and early commitment stage of adipocyte differentiation (R-Diff).

**Figure 2:**
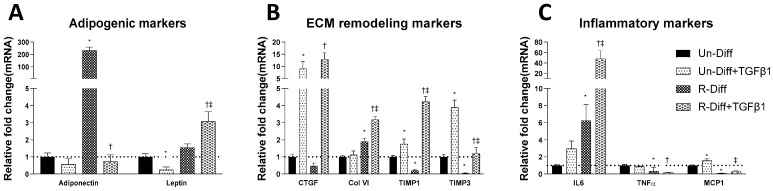
Effect of TGFβ1 on adipogenic (**A**), ECM remodeling (**B**) and inflammatory (**C**) markers on Preadipocytes (Un-Diff) and Commitment stage of adipocyte differentiation (R-Diff). The result is a representative result of 2 independent experiments. Data expressed as Mean ± SD. Two-way ANOVA with Sidak’s multiple comparison test used to compare the effect of TGFβ1 on Un-Diff and R-Diff. *p*-value < 0.05 * vs. Un-Diff, † vs. R-Diff. ‡ vs. Un-Diff + TGFβ1.

**Figure 3:**
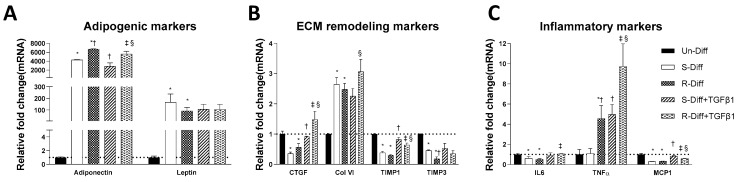
Effect of TGFβ1 on adipogenic (**A**), ECM remodeling (**B**), and inflammatory (**C**) markers on Terminal differentiated adipocytes with S-diff and R-Diff. The result is a representative result of 3 independent experiments. Data expressed as Mean ± SD. One-way ANOVA with Tukey’s multiple comparison test was used to compare Un-Diff, S-Diff, and R-Diff. Two-way ANOVA with Sidak’s multiple comparison test was used to compare the effect of TGFβ1 on S-Diff and R-Diff. *p*-value < 0.05 * vs. Un-Diff, † vs. S-Diff. ‡ vs. R-Diff, § vs. S-Diff + TGFβ1.

The adequate differentiation of adipocytes in our experiment was indicated by the presence in differentiated cells of a marked increase in adiponectin mRNA in both early (>100 fold) and terminal (>1000 fold) differentiation stages of cultured adipocytes (Figure 1, Figure 2A and Figure 3A). Increased leptin mRNA was also observed in mature adipocytes (>100 fold) but was not increased in the commitment phase of adipocyte differentiation (Figure 2A and Figure 3A). In comparing the effect of the differentiation mixes on adipogenesis, the significant increase in adiponectin mRNA and the increased ORO staining in R-Diff cells indicated a greater preponderance of mature adipocytes in R-Diff media (Figure 1).

**Figure 4:**
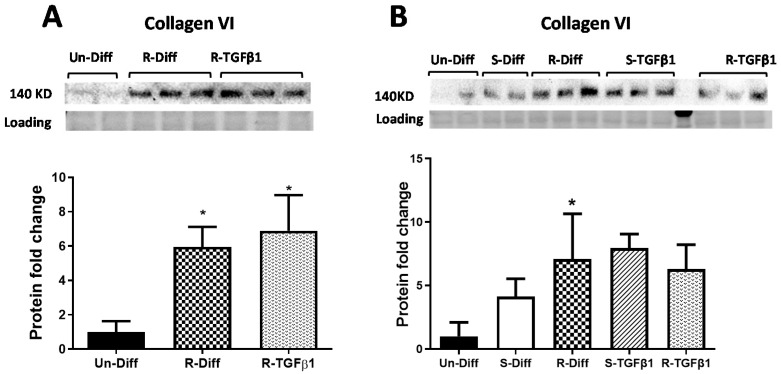
Effect of TGFβ1 on Collagen VI protein, (**A**) Commitment stage of adipocyte differentiation: The result is a representative result of 2 independent experiments. Data expressed as Mean ± SD. One-way ANOVA with Tukey’s multiple comparison test was used to compare the effect of TGFβ1 on Un-Diff and R-Diff. *p*-value < 0.05 * vs. Un-Diff, vs. R-Diff. (**B**) Mature adipocytes: The result is a representative result of 3 independent experiments. Data expressed as Mean ± SD. One-way ANOVA with Tukey’s multiple comparison test was used to compare Un-Diff, S-Diff, and R-Diff. Two-way ANOVA with Sidak’s multiple comparison test was used to compare the effect of TGFβ1 on S-Diff and R-Diff. *p*-value < 0.05 * vs. Un-Diff, vs. S-Diff. vs. R-Diff, vs. S-Diff + TGFβ1. The levels of collagen VI protein, visualized by Western blot, were normalized to total protein loading obtained from stain-free imaging and shown in the histogram.

Adipocytes at commitment and terminal phases of adipocyte differentiation expressed significantly more collagen VI mRNA (Figure 2B and Figure 3B). Similar increases in collagen VI protein were observed in adipocytes differentiated with R-Diff but not in the S-Diff (Figure 4). Surprisingly, despite such an increase in gene expression of collagen VI, its regulators CCN2/CTGF, TIMP1, and TIMP3 were down-regulated at both the commitment and terminal phase of adipocyte differentiation (Figure 2B and Figure 3B). A comparison of the regulators of ECM remodeling markers between S-Diff and R-Diff treated cells showed there were no differences in gene expression of collagen VI, CCN2/CTGF, and TIMP1 mRNA, but TIMP3 mRNA expression was decreased in R-Diff cells compared to S-Diff cells (*p* < 0.05) (Figure 3B).

There were distinct changes in inflammatory markers during adipocyte differentiation. Early in the commitment stage of adipocyte differentiation, the gene expression of tumor necrosis factor-α (TNFα) was significantly decreased, and interleukin-6 (IL6) was increased (*p* < 0.05 compared to undifferentiated preadipocytes). This pattern was reversed in the terminal differentiation stage, where TNFα was increased, and IL6 was decreased. But monocyte chemoattractant protein-1 (MCP1) was decreased in both the commitment and terminal stage of adipocyte differentiation.

### 3.2. TGFβ1 Treatment Affects Both the Synthesis and Degradation of Fibrosis Differently at Certain Stages of Adipocyte Differentiation

TGFβ1 treatment significantly decreased adiponectin mRNA in both the commitment (>300 fold) and terminally differentiated cells [S-Diff (~33%) or R-Diff (~20%)] with a decrease in adiponectin indicating the degree of inhibition of mature differentiation. The inhibitory effect of TGFβ1 on adipogenesis at the commitment stage was further substantiated by the marked reduction in the oil red O staining of adipocytes on days 3, 6, and 10 of TGFβ1 treatment (Figure 1). In contrast, TGFβ1 markedly upregulated leptin mRNA in cells at the commitment stage but not in terminally differentiated adipocytes (Figure 2A and Figure 3A). In preadipocyte, TGFβ1 did not change adiponectin mRNA but significantly decreased leptin mRNA (Figure 2A).

TGFβ1 increased the gene expression of CCN2/CTGF and TIMP1 in preadipocytes when added during the commitment stage, but when added to terminally differentiated adipocytes, there was no difference seen between the S-Diff and R-Diff media. The increases in TIMP3 and collagen VI occurred with TGFβ1 treatment of preadipocytes and during the commitment stage but not in terminally differentiated adipocytes (Figure 2B and Figure 3B). An increase in collagen VI protein was not detected despite an increase in collagen mRNA in the commitment stage induced by TGFβ1 treatment (Figure 2B and Figure 4A). Consistent with the effect of TGFβ1 on collagen VI mRNA in terminally differentiated adipocyte, no significant change in collagen VI protein was observed (Figure 4B).

In preadipocytes, the effect of TGFβ1 on inflammatory marker gene expression was minimal, with no changes in IL6 and TNFα and only a small increase in MCP1 (~50%) (Figure 2C). In terminally differentiated adipocytes with R-Diff, TGFβ1 treatment upregulated gene expression of IL6, TNFα, and MCP1. But in the terminally differentiated adipocyte with S-Diff, TGFβ1 treatment did not show changes in IL6, although similar changes to R-Diff cells were observed for both TNFα and MCP1 (Figure 2C and Figure 3C). This contrasted with the changes during the commitment stage of adipocyte differentiation, where TGFβ1 downregulated TNFα mRNA and increased MCP1 and IL6 mRNA (Figure 2C and Figure 3C).

## 4. Discussion

TGFβ1 produced minimal changes in the adipogenic and inflammatory markers but significantly upregulated fibrotic makers in preadipocytes. In the commitment and terminal stages of adipocyte differentiation, adiponectin was decreased while IL6 and MCP1 were increased. Interestingly leptin, another adipogenesis marker, was increased during the commitment stage, but TGFβ1 had no effect during the terminal stage of adipocyte differentiation. There was an expected increase in fibrotic markers with TGFβ1 treatment at both the commitment and terminal stages of adipocyte differentiation, with a greater increase occurring in the commitment stage. TGFβ1 caused a decrease in the commitment stage of TNFα but an increase in the terminal stage of adipocyte differentiation where increased TNFα would be consistent with an insulin-resistant state caused by the high insulin used in the media.

Sparks et al. showed a decrease in adiponectin mRNA after TGFβ1 treatment of adipocytes at the commitment stage [22], as we confirmed in this study, but we also showed an effect at the terminal stage of adipocyte differentiation. In preadipocytes, the effect of TGFβ1 on adiponectin mRNA was unremarkable and could be due to a very low capacity to express adiponectin in these cells. Also, a study by Choy et al. showed decreased TGFβ receptor signaling results in accelerated adipogenesis [23]. Therefore, it is likely that the less expression of TGFβ receptors in preadipocytes resulted in an insignificant effect in adipogenesis at this stage in this study. Other studies showed a decrease in leptin mRNA with TGFβ1 treatment that was consistent with the change in adiponectin mRNA [24], but in our study, we found leptin mRNA had increased with TGFβ1 treatment of adipocytes during the commitment stage.

During adipocyte differentiation, increases in collagen VI mRNA and protein were observed in adipocytes at commitment and terminal stages of differentiation, consistent with other studies where collagen VI protein was shown to be elevated by the 4th day of adipocyte differentiation and was sustained throughout the maturation phase [25]. However, this increase in collagen did not correlate with an expected increase in profibrotic factors instead we observed a decrease in CCN2/CTGF mRNA which was contrary to the expected effect of TGFβ1 [12]. This suggested that the increase in collagen VI during the physiological differentiation of adipocytes may be regulated through other non TGFβ1 mediated pathways. For example, in addition to the roles of TIMP1 and TIMP3 in ECM remodeling, they also have an inhibitory effect on adipocyte differentiation [26,27]. Our observation of a decline in TIMP1 and TIMP3 at both the commitment and terminal stages of adipocyte differentiation would be consistent with their inhibitory role in adipocyte differentiation.

Generally, TGFβ suppresses MMPs and induces TIMPs to shift ECM balance towards a synthetic phenotype [28]. In our study, TGFβ1 treatment upregulated collagen VI mRNA along with its regulators, i.e., CCN2/CTGF, TIMP1, and TIMP3 mRNA in the commitment stage of adipocytes indicating that TGFβ1 was driving fibrotic changes that would be needed for tissue expansion. The TGFβ1-induced increase of CCN2/CTGF, TIMP1, and TIMP3 in our study was consistent with the studies in other tissues [12,13,29,30]. However, in adipocytes, an enhancing effect of TGFβ1 on TIMP3 mRNA has not been reported before. Despite the known anti-fibrotic function of TIMP3 [31,32,33], TGFβ1 treatment upregulated TIMP3, which would be inhibitory to the profibrotic role of TGFβ1. The effect of TGFβ1 did not increase the mRNA levels of collagen VI or of TIMP3 in terminally differentiated cells which could be due to the absence of a requirement for ECM synthesis in mature adipocytes.

Increased inflammatory responses have been reported to be associated with the inhibition of adipogenesis [34,35,36] but seem to be essential for adipose tissue expansion [37]. In accord with this, we also observed lower levels of MCP1 and IL6 mRNA in the terminal stage of adipocyte differentiation, suggesting that the decrease in inflammatory changes may facilitate adipocyte differentiation. Moreover, inhibition of adipogenesis with TGFβ1 treatment coincides with an increase in IL6 and MCP1 at both commitment and terminal stages of adipocyte differentiation which was consistent with a similar effect of TGFβ1 on human corneal epithelium cells [38].

Other in vitro studies have shown an inhibitory effect of exogenous TNFα on adipocyte differentiation [36,39,40,41]. However, we found increased levels of TNFα mRNA in terminally differentiated adipocytes which seemed to be contradictory at first but fitted with the increasing evidence of the role of pro-inflammatory requirements for adipocyte differentiation and expansion [42,43,44,45]. This was consistent with the in vivo study by Wernstedt, Asterholm, et al., where reduced TNFα expression led to the inhibition of adipocyte differentiation [37]. Similarly, TGFβ1 treatment of adipocytes at the commitment stage downregulated TNFα and inhibited adipocyte differentiation. In contrast, TGFβ1 treatment of adipocytes at the terminal differentiation stage upregulated TNFα despite its inhibitory effect on adipocyte differentiation. These findings were consistent with Li et al. [38] but contrasted with other studies [46]. The dependence on the stage of adipocyte maturation for an effect of TGFβ1 on TNFα may explain the contrasting findings of a stimulatory or an inhibitory effect of TGFβ1 on TNFα, but it could also be due to the high insulin concentration used in the media required for differentiation, but this requires further investigations.

TGFβ1 has been shown to upregulate the gene expression of CCN2/CTGF, TIMP1, and TIMP3 in adipocytes and in other cells [12,13,19,29], but here we report that the effect of TGFβ1 treatment on these markers was dependent upon the stage of 3T3L1 adipocyte differentiation. Although a study by Choy et al. found a decrease in TGFβ receptors during adipocyte differentiation [23], the effect of TGFβ1 was observed in mature adipocytes in this study. This shows that even though receptors are decreased in mature adipocytes, they are sufficient to have an inhibitory effect. It is further substantiated by our previous findings, which showed that the ability to stimulate CCN2 is preserved despite a reduction in TGFβ receptors [19]. This is a novel finding which has not been appreciated previously. We clearly demonstrated that TGFβ1 significantly increased all the profibrotic gene makers for ECM remodeling (collagen VI, CCN2/CTGF, TIMP1) at all stages of adipocyte differentiation.

Limitations of this study are that in undifferentiated cells, insulin was not present and would not have increased TGFβ receptors. During differentiation, insulin was present but was removed after terminal differentiation. Although insulin was not present during the TGFβ stimulation of mature adipocytes, they had been exposed to high insulin during the commitment stage. Although unlikely, this may be a confounding influence on the results and could be a cause for insulin resistance occurring in cells incubated with high insulin.

## 5. Conclusions

This study has demonstrated a distinct effect of TGFβ1 on adipogenic, ECM remodeling, and inflammatory markers at different stages of adipocyte differentiation. However, in general, TGFβ1 inhibited the adipogenic markers and upregulated ECM and Inflammatory markers but the stimulation of TIMPs at different stages of development by TGFβ appeared to have a modulation effect on the stimulation of ECM.

## Figures and Tables

**Table 1:** Primers used in this study.

Primers	Forward	Reverse
Adiponectin	5′-CGACACCAAAAGGGCTCAGG-3′	5′-ACGTCATCTTCGGCATGACT-3′
Leptin	5′-GCTGCAAGGTGCAAGAAGAAG-3′	5′-TAGGACCAAAGCCACAGGAAC-3′
MCP1	5′-CACTCACCTGCTGCTACTCA-3′	5′-GCTTGGTGACAAAAACTACAGC-3′
IL6	5′-TCCTCTCTGCAAGAGACTTCC-3′	5′-TTGTGAAGTAGGGAAGGCCG-3′
TNFα	5′-GACCCTCACACTCACAAACCA-3′	5′-ACAAGGTACAACCCATCGGC-3′
Collagen VI	5′-GAACTTCCCTGCCAAACAGA-3′	5′-CACCTTGTGGAAGTTCTGCTC-3′
CCN2/CTGF	5′-GAGTGTGCACTGCCAAAGATG-3′	5′-TCCAGGCAAGTGCATTGG T-3′
TIMP1	5′-CACAAGTCCCAGAACCGC-3′	5′-GGATTCCGTGGCAGGC-3′
TIMP3	5′-CTTCTGCAACTCCGACATCGTGAT-3′	5′-CAGCAGGTACTGGTACTTGTTGAC-3′
NoNo	5′-TGCTCCTGTGCCACCTGGTACTC-3′	5′-CCGGAGCTGGACGGTTGAATGC-3′

## Data Availability

Not applicable.
